# Nomogram for Predicting Overall Survival and Assessing the Survival Benefit of Adjuvant Treatment in pT1-2N0M0 Triple-Negative Breast Cancer: A Surveillance, Epidemiology, and End Results-Based Study

**DOI:** 10.3389/fonc.2021.663621

**Published:** 2022-02-25

**Authors:** Qixin Mao, Shanqing Liu, Minhao Lv, Yadong Sun, Chongjian Zhang, Lianfang Li

**Affiliations:** Department of Breast Surgery, Affiliated Cancer Hospital of Zhengzhou University, Zhengzhou, China

**Keywords:** breast cancer, prognosis, nomogram, survival, adjuvant treatment

## Abstract

**Background:**

Accurate survival prediction of triple-negative breast cancer (TNBC) is essential in the decision-making of adjuvant treatment. The aim of this prospective study was to develop a nomogram that predicts overall survival and assists adjuvant treatment formulation.

**Methods:**

A total of 16,977 patients with pT1-2N0M0 TNBC between 2010 and 2015 from the SEER database were enrolled. Independent prognostic factors associated with overall survival (OS) were identified using univariate and multivariate Cox regression hazards method and utilized to compose the nomogram. The survival benefit of adjuvant treatment on OS were analyzed after stratification by nomogram sum-score.

**Results:**

Patients were randomized 7:3 into the training and validation cohorts. Multivariate analysis revealed that age at diagnosis, grade, tumor size, laterality, and mastectomy type were independent prognostic factors of OS and were integrated to develop a nomogram for predicting prognosis. Patients were stratified into 3 prognostic subgroups according to the sum-score of our nomogram. There were no significant differences found in OS between surgery alone and other adjuvant treatment strategies in low risk group. In moderate risk group, patients receiving chemotherapy or the combination of chemotherapy and radiotherapy showed better OS than those receiving surgery alone or radiotherapy alone. For patients in high risk group, the combination of chemotherapy and radiotherapy could maximally improve the overall survival rate of patients.

**Conclusion:**

A novel nomogram for OS prediction and risk stratification in patients with pT1-2N0M0 TNBC was developed. This cohort study reveals the prognostic roles of different adjuvant treatment strategies in subgroups, which may provide a reference for the decision-making of postoperative treatment, eventually improving prognosis for individual patients.

## Introduction

Breast cancer (BC) is the most commonly diagnosed malignant tumor in women, with an estimated incidence of 2,76,000 new cases and 42,000 deaths annually in the United States alone (1). Over the past two decades, the incidence rate of stage I breast cancers has increased dramatically, largely due to the early detection of non-palpable BC associated with mammography screening (2, 3). Patients with small node-negative breast cancer generally have excellent survival outcomes, with ten-year probability of breast cancer-specific mortality exceeding 95% (4). Nevertheless, despite the benign prognosis in patients with small node-negative breast cancer, outcomes may vary by biologic type (5). Triple-negative breast cancer (TNBC), accounting for approximately 15% of all breast cancers cases, shows high malignancy, strong invasiveness, early metastasis, and poorer prognosis, reflecting the vital role of accurate survival prediction and individualized management after surgery for these patients (6, 7).

Whether patients with small node-negative TNBC should receive postoperative adjuvant therapy remains unclear. Previous studies have demonstrated that for T1-2N0M0 TNBC patients, receipt of adjuvant therapy was significantly associated with better overall survival, and delayed initiation of chemotherapy might induce worse outcomes (8, 9). The National Comprehensive Cancer Network (NCCN) guidelines recommend chemotherapy rather than radiotherapy for patients with tumor size larger than 5 mm (10). Nevertheless, a number of studies demonstrated that postoperative radiotherapy could minimize the risk of regional recurrence of triple-negative breast cancer (11). Therefore, the survival benefit of different adjuvant treatments in patients with T1-2N0M0 TNBC needs further investigation.

Accurate prognosis prediction serves as an vital reference factor in the decision-making of individualized treatment. Nomogram, a tool incorporating prognostic risk factors, could visually estimate the survival probability of patients based on a statistical predictive model (12). Therefore, this study aimed to construct a nomogram for predicting the probability of overall survival (OS) of patients with pT1-2N0M0 triple-negative breast cancer to assist the decision-making of postoperative adjuvant treatments.

## Methods

### Data Collection

**Figure 1:**
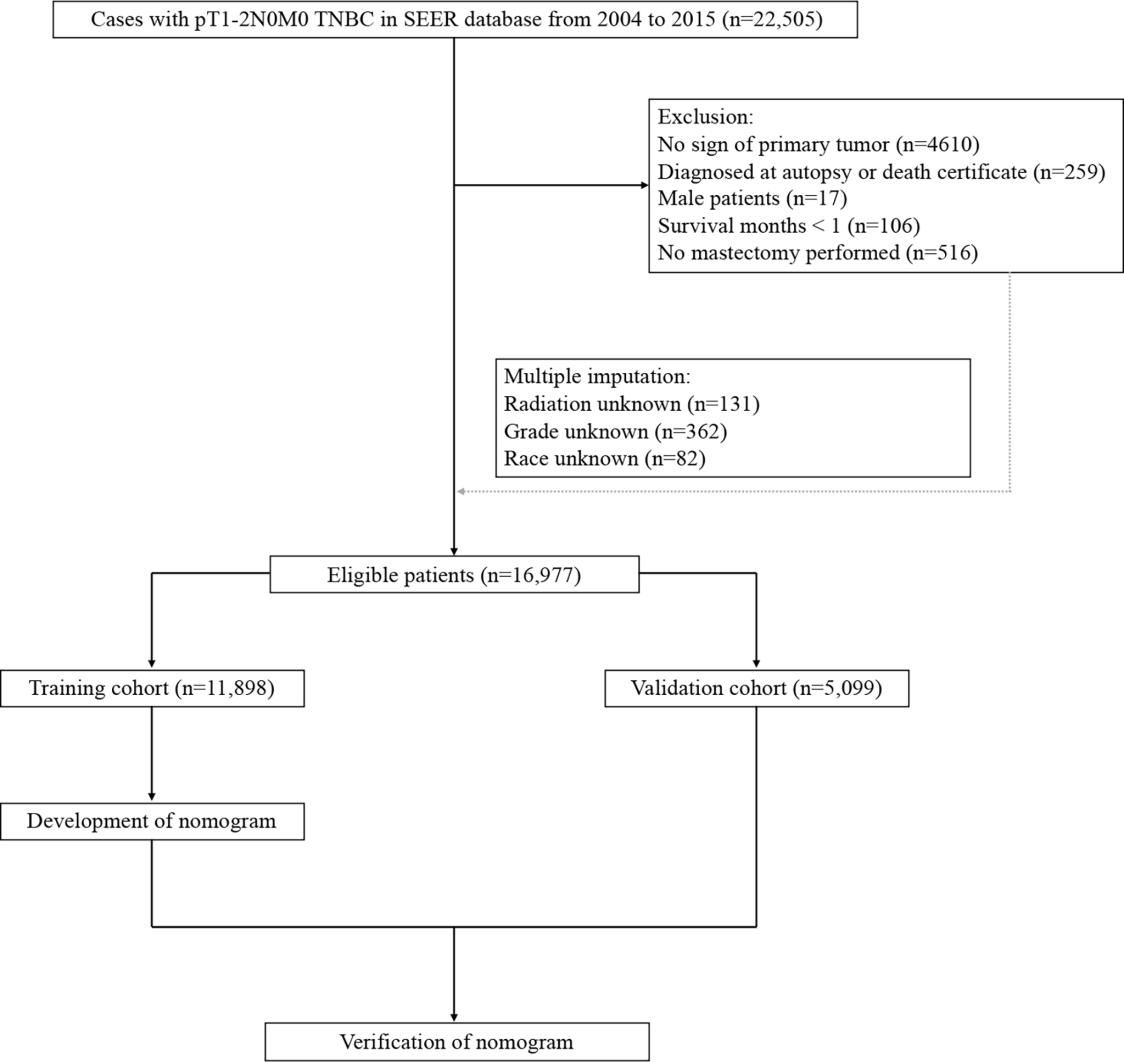
The flowchart.

Using SEER*Stat version 8.3.6.1, the data of eligible TNBC patients registered between 2010 and 2015 were collected from Surveillance, Epidemiology, and End Results (SEER) program, which was the largest publicly available cancer database and provided deidentified information regarding cancer statistics of approximately 28% of the US population. TNBC was defined as ERBB2 negative, progesterone receptor (PR) negative, and estrogen receptor (ER) negative. The following inclusion criteria were utilized to identify analysis cohort: female sex; older than 18 years old; diagnosed with pT1-2N0M0 TNBC; receiving mastectomy with or without adjuvant therapy. Patients diagnosed by autopsy or a death certificate, and those with more than 1 primary malignant neoplasm were excluded. Multiple imputation by chained equations was used to replace missing data as detailed in the flowchart (**Figure 1**).

Before the initiation of this study, a data-use agreement was submitted to the SEER program and an access to the SEER database were officially granted. Demographic and clinicopathological characteristics of eligible patients were extracted for analysis, including age at diagnosis, histological grade, race, year of diagnosis, AJCC Seventh T stage, surgery, chemotherapy, and radiotherapy. The primary endpoint of our study was all-cause mortality.

### Nomogram Construction and Validation

**Figure 2:**
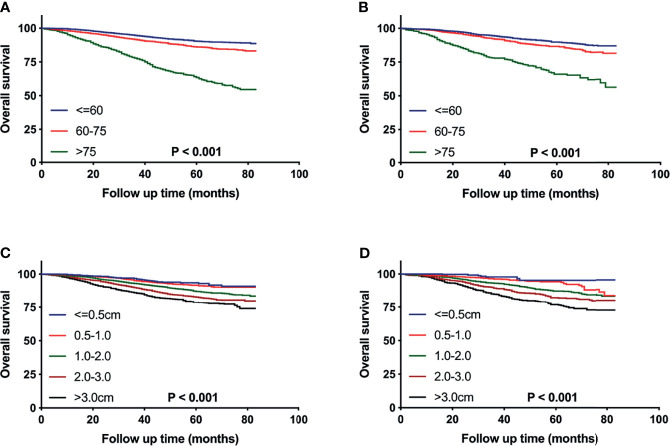
Kaplan–Meier curves comparing OS for pT1-2N0M0 TNBC patients stratified by the cutoff values of age **(A, B)** or tumor size **(C, D)** in the training cohort **(A, C)** and the validation cohort **(B, D)**, respectively.

After patient selection, 16,977 eligible patients were finally included for analysis. These patients were randomized 7:3 to the training cohort for nomogram construction and the validation cohort for nomogram verification, respectively. The optimal cut-off values for age at diagnosis and tumor size with minimum P values for the log-rank test and the highest specificity and sensitivity were determined as 60 and 75 years and 0.5, 1.0, 2.0 and 3.0cm using the X-tile program (Yale University School of Medicine, New Haven, CT, USA). The survival differences were significant stratified by the two variables both in training and validation cohorts (**Figure 2**). All extracted variables were analyzed by univariate analysis, and the variables, which were statistically meaningful (P < 0.05), were taken into multivariable Cox analysis to identify the independent prognostic factors of pT1-2N0M0 TNBC patients. Hazard ratios (HRs) and 95% confidence intervals (CIs) were also calculated. A nomogram based on these statistically significant predictors were then established to estimate a patient of prognosis. The performance of the nomogram was evaluated by calculating concordance index (c-index) (13). Calibration curves were also generated to measure the consistency between the predicted and actual OS at 1, 3, and 5 years in the training and validation cohorts, respectively (14).

Based on the nomogram, sum-scores for individual patients were calculated using the “nomogramFormula” package. The X-tile software was applied to determine the optimal cutoff values for sum-score to classify patients into three risk subgroups.

### Statistical Analysis

The baseline characteristics of patients were described using proportions and frequencies, and compared between different subgroups using the chi-square test. Kaplan–Meier survival curves and log-rank tests were applied to compare overall survival between subgroups. A two-tailed p <0.05 was considered statistically significant. All statistical analyses were carried out with SPSS statistics software (version 21; IBM Corp, Armonk, NY, USA) and R software (version 3.6.1; http://www.r-project.org).

## Results

### Patient Characteristics

A total of 16,977 patients diagnosed with pT1-2N0M0 triple-negative breast cancer from the SEER database met the inclusion and exclusion criteria. Those patients were more apt to be younger (n = 9352), white (n = 12522), with invasive ductal carcinoma (n = 14731), and poorly differentiated (n = 13238). The detailed baseline demographics and clinical characteristics were summarized in **Table 1**.

**Table 1:** Demographics and clinicopathological characteristics of patients with pT1-pT2N0M0 triple-negative breast cancer.

Characteristics	Whole cohort	Training cohort	Validation cohort	P value
**Overall No.**	**16997**	**11898**	**5099**	
**Age (years)**				0.042
<=60	9352 (55.0%)	6482 (54.5%)	2870 (56.3%)	
60-75	5654 (33.3%)	4028 (33.9%)	1626 (31.9%)	
>75	1991 (11.7%)	1388 (11.6%)	603 (11.8%)	
**Ethnicity**				0.481
White	12552 (73.8%)	8818 (74.1%)	3734 (73.2%)	
Black	3152 (18.5%)	2182 (18.4%)	970 (19.1%)	
Others^#^	1293 (7.7%)	898 (7.5%)	395 (7.7%)	
**Year of diagnosis**				0.558
2010-2013	8651 (50.9%)	6038 (50.7%)	2613 (51.2%)	
2014-2016	8346 (49.1%)	5860 (49.3%)	2486 (48.8%)	
**Histology**				0.404
Invasive ductal carcinoma, IDC	14731 (86.7%)	10286 (86.5%)	4445 (87.2%)	
Invasive lobular carcinoma, ILC	834 (4.9%)	598 (5.0%)	236 (4.6%)	
Others	1432 (8.4%)	1014 (8.5%)	418 (8.2%)	
**Laterality**				0.694
Right	8271 (48.7%)	5778 (48.6%)	2493 (48.9%)	
Left	8726 (51.3%)	6120 (51.4%)	2606 (51.1%)	
**Grade**				
Well-differentiated	472 (2.8%)	330 (2.8%)	142 (2.8%)	0.936
Moderately differentiated	3174 (18.7%)	2220 (18.7%)	954 (18.7%)	
Poorly differentiated	13238 (77.9%)	9272 (77.9%)	3966 (77.8%)	
Undifferentiated	113 (0.7%)	76 (0.6%)	37 (0.7%)	
**Tumor size (cm)**				0.389
<=0.5	1027 (6.1%)	713 (6.0%)	314 (6.2%)	
0.5-1.0	2491 (14.7%)	1743 (14.6%)	748 (14.7%)	
1.0-2.0	6328 (37.2%)	4397 (37.0%)	1931 (37.9%)	
2.0-3.0	4443 (26.1%)	3162 (26.6%)	1281 (25.1%)	
>3.0	2708 (15.9%)	1883 (15.8%)	825 (16.1%)	
**Surgery**				0.744
Partial mastectomy	10920 (64.2%)	7663 (64.4%)	3257 (63.9%)	
Total mastectomy	4642 (27.3%)	3229 (27.1%)	1413 (27.7%)	
Radical mastectomy	1435 (8.5%)	1006 (8.5%)	429 (8.4%)	
**Adjuvant therapy**				0.728
None	3005 (17.7%)	2105 (17.7%)	900 (17.7%)	
Chemotherapy	2041 (12.0%)	1434 (12.1%)	607 (11.9%)	
Radiation	5714 (33.6%)	3969 (33.4%)	1745 (34.2%)	
Both	6237 (36.7%)	4390 (36.9%)	1847 (36.2%)	

^#^American Indian/AK Native, Asian/Pacific Islander.

Overall, the median follow-up time was 42 months (range, 0 to 83 months). Patients were randomized 7:3 to the training cohort (n = 11898) and the validation cohort (n = 5099). 1197 deaths occurred in the training cohort and 518 in the validation cohort. The 1-, 3-, 5-year, and 10-year overall survival rates were 98.0%, 95.0%, 91.6%, 85.7% in the training set and 98.4%, 95.1%, 91.7%, 85.8% in the validation set, respectively. There were no significant differences between the two cohorts in all factors included (p > 0.05, **Table 1**), reflecting that the two sets were statistically comparable.

### Risk Analysis of Death in Patients With pT1-2N0M0 TNBC

Univariate and multivariate analysis were performed to further investigate the prognostic factors of death in pT1-2N0M0 TNBC patients. **Table 2** showed that in the training cohort, the independent risk variables for overall survival were age at diagnosis (> 75 years: HR 5.267; 95% CI 4.692-5.911; p < 0.001), tumor size (> 3.0cm: HR 4.500; 95% CI 3.289-6.158; p < 0.001), differentiated grade (poorly differentiated: HR 1.627; 95% CI 1.150-2.303; p < 0.001), laterality (left: HR 1.140; 95% CI 1.109-1.278; p < 0.001), and surgery (total mastectomy: HR 1.117; 95% CI 1.003-1.244; p < 0.043).

**Table 2:** Univariate and multivariate analysis for the prognostic characteristics of OS.

Characteristics	Univariate analysis			Multivariate analysis		
	HR^1^	95%CI^2^	P value	HR	95%CI	P value
**Age(years)**						
<=60	Ref			Ref		
60-75	1.514	1.353-1.695	0.000	1.775	1.583-1.990	0.000
>75	4.596	4.101-5.150	0.000	5.267	4.692-5.911	0.000
**Ethnicity**						
White	Ref					
Black	1.063	0.944-1.196	0.314			
Others^#^	0.616	0.494-0.768	0.000			
**Year of diagnosis**						
2010-2013	Ref					
2014-2016	0.877	0.765-1.006	0.051			
**Histology**						
Invasive ductal carcinoma, IDC	Ref					
Invasive lobular carcinoma, ILC	1.025	0.829-1.268	0.819			
Others	1.020	0.862-1.206	0.818			
**Laterality**						
Right	Ref			Ref		
Left	1.118	1.018-1.228	0.020	1.140	1.109-1.278	0.000
**Grade**						
Well-differentiated	Ref			Ref		
Moderately differentiated	1.434	1.001-2.054	0.049	1.416	0.988-2.029	0.058
Poorly differentiated	1.653	1.170-2.334	0.004	1.627	1.150-2.303	0.006
Undifferentiated	3.036	1.783-5.171	0.000	2.904	1.702-4.955	0.000
**Tumor size(cm)**						
<=0.5	Ref			Ref		
0.5-1.0	1.339	0.956-1.876	0.089	1.346	0.961-1.886	0.084
1.0-2.0	2.143	1.577-2.911	0.000	2.230	1.639-3.034	0.000
2.0-3.0	3.009	2.214-4.091	0.000	3.296	2.418-4.494	0.000
>3.0	4.115	3.019-5.610	0.000	4.500	3.289-6.158	0.000
**Surgery**						
Partial mastectomy	Ref			Ref		
Total mastectomy	1.182	1.063-1.314	0.002	1.117	1.003-1.244	0.043
Radical mastectomy	1.511	1.307-1.747	0.000	1.223	1.055-1.416	0.007

^1^HR Hazard ratio; ^2^CI confidence interval; ^#^American Indian/AK Native, Asian/Pacific Islander.

### Development and Validation of Nomogram for OS

**Figure 3:**
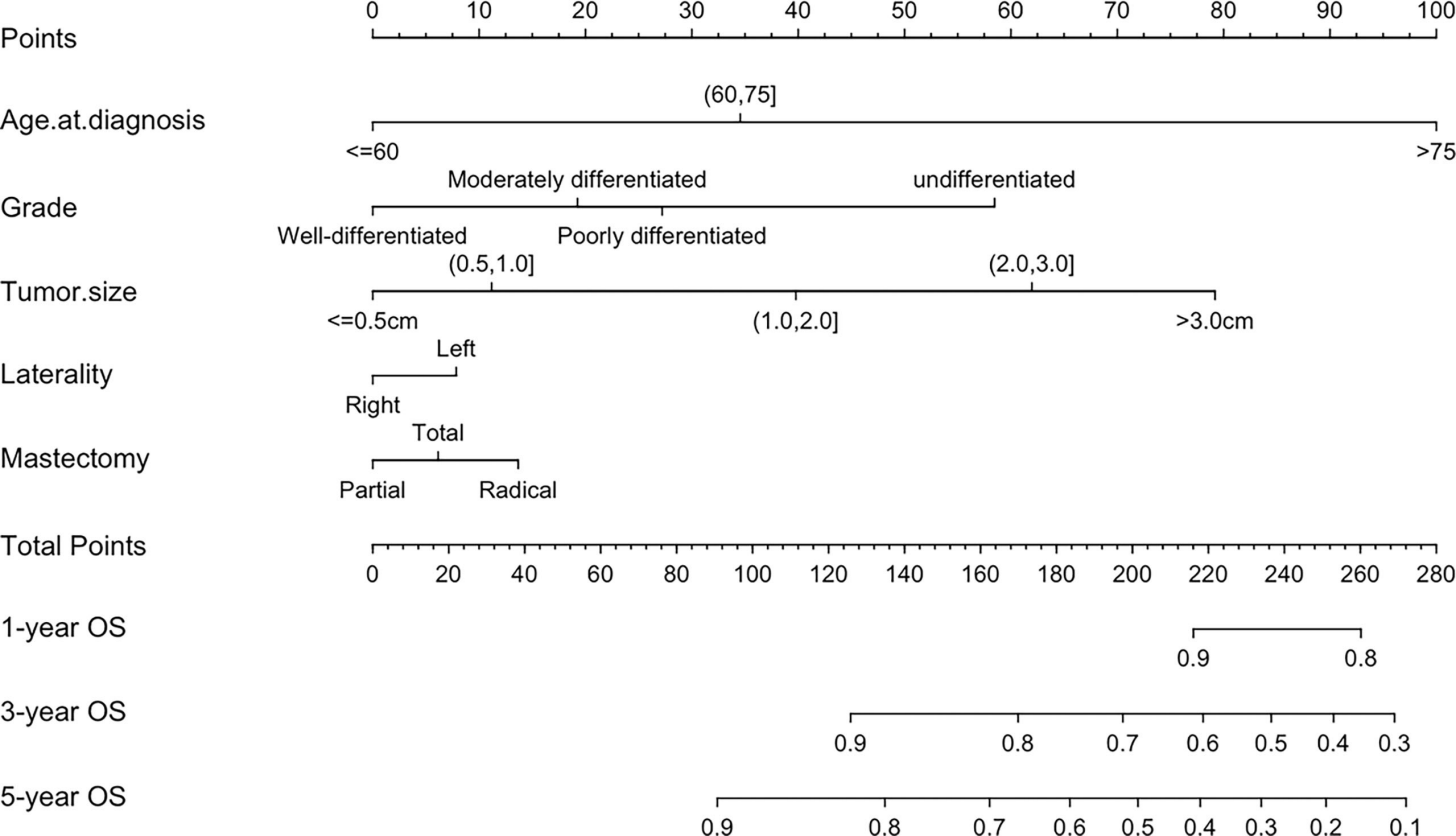
Nomogram for predicting OS in patients with pT1-2N0M0 TNBC.

**Figure 4:**
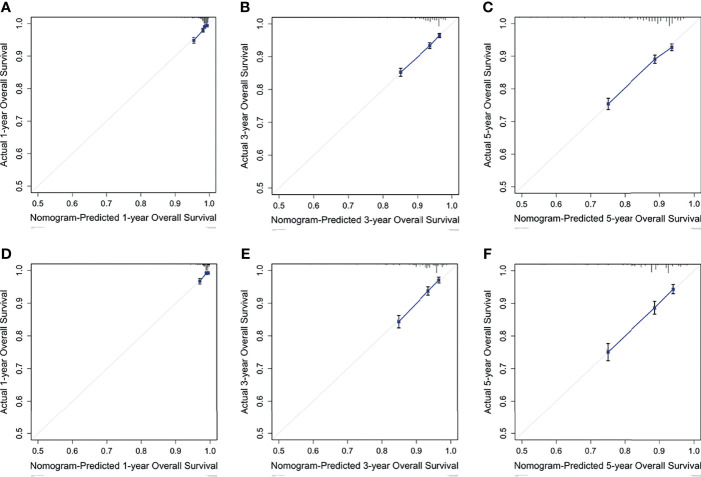
Calibration curves predict the nomogram-estimated and actual 1-, 3-, and 5-year OS rates in the training **(A-C)** and validation **(D-F)** cohorts.

Based on the results of multivariate analysis, a nomogram was formulated to estimate the 1-, 3-, and 5-year OS for patients with pT1-2N0M0. The C-indexes of the model were 0.705 (95% CI: 0.688-0.721) and 0.703 (95% CI: 0.677-0.726) in the training and validation sets (**Figure 3**). We utilized beta-coefficients to assign scores to variables. By calculating the sum-scores, the probability of all-cause death could be predicted for individual patients. In our OS-predicting model, age at diagnosis and tumor size were the largest contributors to OS estimation. Calibration curves of the nomogram indicated that the predicted survival rates were almost consistent with the actual observations (**Figure 4**).

### Risk Stratification Based on Nomogram Sum-Score

**Figure 5:**
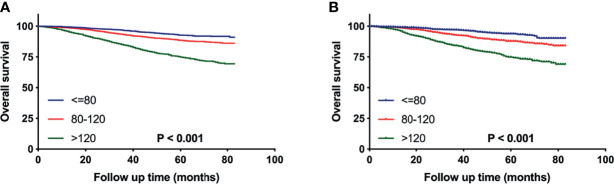
Overall survival of pT1-2N0M0 TNBC patients stratified by nomogram sum-score in the training **(A)** and validation **(B)** cohort.

The X-tile analysis was utilized to establish a risk stratification system according to the nomogram sum-score. Patients with pT1-2N0M0 TNBC were classified into 3 subgroups based on their sum-scores. As shown in **Figure 5**, stratification into different prognostic groups allowed significant discrepancy between Kaplan-Meier plots for overall survival outcomes in both training and validation cohorts. In the training set, as the sum-scores increased, the 5-year overall survival rate declined significantly: 92.7% in low risk group, 88.5% in moderate risk group, and 74.9% in high risk group (p < 0.01).

### Subgroup Analysis Stratified by Sum-Score

**Figure 6:**
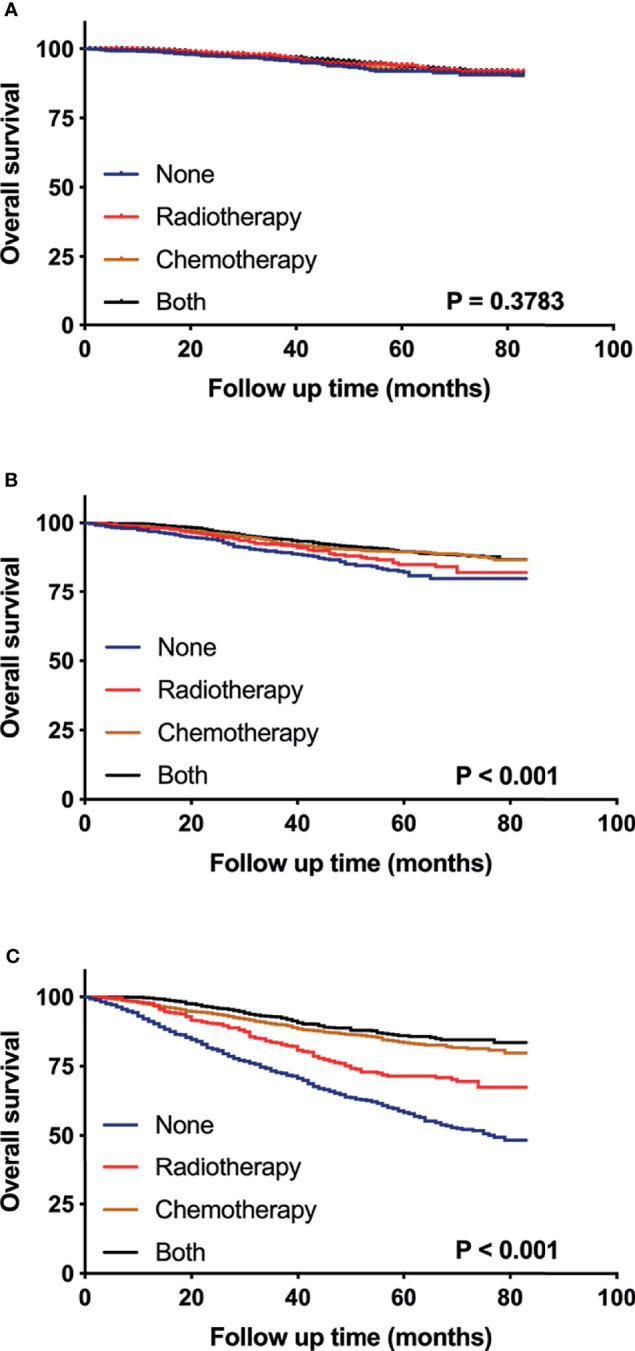
Kaplan–Meier curves compare the survival effects of different adjuvant treatment strategies for pT1-2N0M0 TNBC patients in low-risk **(A)**, moderate-risk **(B)**, and high-risk subgroups **(C)**.

To evaluate the survival benefit of post-surgery adjuvant treatments (i.e. surgery alone, radiotherapy, chemotherapy, and the combination of radiotherapy and chemotherapy) in three risk groups respectively, we generated the Kaplan-Meier curves (**Figure 6**) and calculated hazard ratios for each adjuvant therapy (**Table 3**). For patients in low risk group, no significant differences were found in OS between surgery alone and other adjuvant treatments (**Figure 6A**). Nevertheless, inconsistent with the result of low-risk group, in moderate risk group, patients who received chemotherapy (HR 0.598; 95% CI 0.469-0.761; p < 0.001) or the combination of radiotherapy and chemotherapy (HR 0.538; 95% CI 0.423-0.683; p < 0.001) showed significantly better OS compared those receiving surgery alone or radiotherapy alone (**Figure 6B**). For patients in high risk group (**Figure 6C**), the combination of radiotherapy and chemotherapy (HR 0.266; 95% CI 0.217-0.325; p < 0.001) was associated with the most improved OS compared with surgery alone, followed by chemotherapy (HR 0.319; 95% CI 0.271-0.374; p < 0.001), and radiotherapy (HR 0.572; 95% CI 0.476-0.687; p < 0.001).

**Table 3:** Univariate analysis evaluating the effect of adjuvant treatment strategies stratified by subgroups.

Adjuvant treatment	Low risk group		Moderate risk group		High risk group	
	HR (95% CI)	P value	HR (95% CI)	P value	HR (95% CI)	P value
**Surgery alone**	Ref		Ref		Ref	
**Radiotherapy**	0.780 (0.513-1.186)	0.245	0.791 (0.566-1.105)	0.169	0.572 (0.476-0.687)	0.000
**Chemotherapy**	0.920 (0.638-1.328)	0.656	0.598 (0.469-0.761)	0.000	0.319 (0.271-0.374)	0.000
**Both**	0.756 (0.535-1.067)	0.111	0.538 (0.423-0.683)	0.000	0.266 (0.217-0.325)	0.000

## Discussion

In the present study, we analyzed a cohort of 16977 patients with pT1-2N0M0 triple-negative breast cancer to construct and internally validate practical nomograms for 1-, 3-, and 5-year OS prediction. The availability of tool for predicting the prognosis of diseases accurately may have significant reference role in clinical management. In general, the traditional AJCC stage system plays an important role in predicting survival outcomes and influencing the therapeutic decision-making. However, considering the variability in tumor biology and clinicopathological features, the prognoses of patients within the same AJCC stage remain heterogeneous (15). Our nomograms integrate various clinical risk factors to further explore the prognosis of patients with pT1-2N0M0 TNBC, which may have potential implications for cancer management.

In the univariate and multivariate analyses, age at diagnosis was identified as an significantly independent risk factor for pT1-2N0M0 TNBC patients, as was tumor diameter, grade, laterality, and surgery type. This finding is highly consistent with the results of previous studies on the prognostic factors for TNBC (16–18). Notably, breast cancer in female is more likely to occur in the left breast than in the right (19). This finding has gained the attention of many researchers and numerous hypotheses have been proposed to account for the left-side dominance of breast cancer, but none have been uniformly accepted or confirmed (20, 21). In this study, we found that left-side dominance was also a risk factor for pT1-2N0M0 TNBC, which has not been reported previously. This finding may be worthy of verification through large-scale breast cancer studies on different regions. These variables could be accessed easily and contribute to the individualized prognosis prediction. Nevertheless, the AJCC stage system is based solely on tumor diameter to further stratify patients with pT1-2N0M0 and fails to take into account of other risk factors. Thus, in this study, we incorporated these factors to construct nomogram for OS prediction. Calibration curves showed excellent consistency between the predicted survival rate and actual observation. Notably, the C-indexes of nomograms were acceptable both in the training and validation cohorts, but could be further improved if more important risk factors were incorporated, such as potential serum biomarkers and vascular infiltration.

Results from researches exploring the survival benefit of adjuvant treatment in small node-negative triple-negative breast cancer have been far from conclusive (22). Although current NCCN guideline recommend chemotherapy for patients with tumors larger than 5mm (10), to our knowledge, the clinical evidence supporting this recommend remains limited. Some studies have reported better prognosis for pT1-2N0M0 TNBC patients who received adjuvant chemotherapy, while other studies failed to prove the survival benefit of adjuvant chemotherapy (8, 23, 24). In terms of adjuvant radiotherapy, a prospective randomized controlled multi-center study demonstrated that compared to chemotherapy alone, the combination of standard adjuvant chemotherapy and radiotherapy was associated with significant improvements in overall survival and recurrence-free survival in patients with early-stage TNBC after surgery (25). While, other studies failed to find this association (22). Hence, the survival benefit of postoperative adjuvant treatment in small node-negative TNBC should be further explored.

Accurate identification of patients at a high risk of mortality may help the decision-making of postoperative adjuvant therapy. In the present study, patients with pT1-2N0M0 TNBC after surgery were classified into 3 subgroups base on their accumulated sum-scores using our nomograms. The role of different adjuvant treatments in OS was investigated in subgroups, respectively. For patients in low risk group, the survival outcomes stratified by adjuvant treatments were comparable, indicating that the benefit to risk ratio should be assessed comprehensively when adjuvant therapies were considered. Notably, receiving chemotherapy was associated with improved overall survival in moderate risk group, indicating that clinicians should consider routine postoperative chemotherapy for patients with moderate risk of all-cause mortality. In addition, the combination of chemotherapy and radiotherapy could maximally improve the overall survival rate of patients in high risk group. Thus, we strongly recommended the combination of chemotherapy and radiotherapy for patients with high risk of all-cause mortality, which should be further confirmed by prospective studies.

There were several limitations of the current study that should be acknowledge. First, the SEER database lacks information on serum biomarkers, vascular infiltration, and surgical margin. Failing to incorporate these important prognostic parameters may influence the effectiveness of our nomograms to some extent. For the same reason, we were unable to evaluate the influence of different adjuvant treatment strategies on the prognosis of TNBC patients, which may have led to unconvincing results. Second, the data of the training and validation sets originated from the same database and restricted to the United States, so external verifications of our findings were needed in other counties to enhance reliability. Third, our study was also limited by its retrospective nature and selection biases. However, this population-based design with a considerable sample size has ensure the robustness of our results to some degree. In the future, further studies were expected to collect prospective data, increase sample size, and incorporate more prognostic parameters to improve the accuracy and versatility of the nomogram.

## Conclusion

In summary, a novel nomogram for OS prediction and risk stratification in patients with pT1-2N0M0 TNBC was developed. Evaluating the survival benefit of different adjuvant therapies remains a major concern in the postoperative treatment of TNBC. This cohort study reveals the prognostic roles of different adjuvant treatment strategies in subgroups, which may provide a reference for the decision-making of postoperative treatment, eventually improving prognosis for individual patients.

## Data Availability Statement

The original contributions presented in the study are included in the article/supplementary material. Further inquiries can be directed to the corresponding author.

## Ethics Statement

Ethical review and approval was not required for the study on human participants in accordance with the local legislation and institutional requirements. Written informed consent for participation was not required for this study in accordance with the national legislation and the institutional requirements.

## Author Contributions

QM, SL, and LL conceived the concept, designed the research, drafted and reviewed the article. QM, ML, YS, and CZ extracted and analyzed the data and wrote the manuscript. All authors contributed to the article and approved the submitted version.

## Conflict of Interest

The authors declare that the research was conducted in the absence of any commercial or financial relationships that could be construed as a potential conflict of interest.

## Publisher’s Note

All claims expressed in this article are solely those of the authors and do not necessarily represent those of their affiliated organizations, or those of the publisher, the editors and the reviewers. Any product that may be evaluated in this article, or claim that may be made by its manufacturer, is not guaranteed or endorsed by the publisher.
